# MDA-MB-231 Breast Cancer Cells Resistant to Pleurocidin-Family Lytic Peptides Are Chemosensitive and Exhibit Reduced Tumor-Forming Capacity

**DOI:** 10.3390/biom10091220

**Published:** 2020-08-22

**Authors:** Ashley L. Hilchie, Erin E. Gill, Melanie R. Power Coombs, Reza Falsafi, Robert E. W. Hancock, David W. Hoskin

**Affiliations:** 1 Department of Microbiology and Immunology, Dalhousie University, Halifax, NS B3H 4R2, Canada; ashleyhilchie@gmail.com; 2 Department of Microbiology and Immunology, University of British Columbia, Vancouver, BC V6T 1Z4, Canada; erin.gill81@gmail.com (E.E.G.); reza@hancocklab.com (R.F.); bob@hancocklab.com (R.E.W.H.); 3 Department of Biology, Acadia University, 33 Westwood Ave, Wolfville, NS B4P 2R6, Canada; melanie.coombs@acadiau.ca; 4 Department of Pathology, Dalhousie University, Halifax, NS B3H 4R2, Canada; 5 Department of Surgery, Dalhousie University, Halifax, NS B3H 4R2, Canada

**Keywords:** anticancer peptide, breast cancer, cytolysis, peptide-resistance, pleurocidin

## Abstract

Direct-acting anticancer (DAA) peptides are cytolytic peptides that show promise as novel anticancer agents. DAA peptides bind to anionic molecules that are abundant on cancer cells relative to normal healthy cells, which results in preferential killing of cancer cells. Due to the mechanism by which DAA peptides kill cancer cells, it was thought that resistance would be difficult to achieve. Here, we describe the generation and characterization of two MDA-MB-231 breast cancer cell-line variants with reduced susceptibility to pleurocidin-family and mastoparan DAA peptides. Peptide resistance correlated with deficiencies in peptide binding to cell-surface structures, suggesting that resistance was due to altered composition of the cell membrane. Peptide-resistant MDA-MB-231 cells were phenotypically distinct yet remained susceptible to chemotherapy. Surprisingly, neither of the peptide-resistant breast cancer cell lines was able to establish tumors in immune-deficient mice. Histological analysis and RNA sequencing suggested that tumorigenicity was impacted by alternations in angiogenesis and extracellular matrix composition in the peptide-resistant MDA-MB-231 variants. Collectively, these data further support the therapeutic potential of DAA peptides as adjunctive treatments for cancer.

## 1. Introduction

Cancer cell resistance to chemotherapeutic drugs, including targeted therapies, remains an obstacle to the eradication of disseminated tumors. As a result, there remains an unmet need for alternative therapeutic agents for the treatment of cancer. Ideally, such an agent would target cancer cells by a mechanism that differs from that of chemotherapeutic drugs currently on the market. Direct-acting anticancer (DAA) peptides represent an as-yet untapped reservoir of such novel anticancer agents.

Anticancer peptides are small peptides that contain cationic and hydrophobic amino acids, giving them an overall positive charge and amphipathic structure [1,2]. Unlike the cell membranes of normal healthy cells, cancer cell membranes carry a net negative charge due to an abundance of anionic phospholipids and proteoglycans [3], which are thought to lead to the selective attraction of anticancer peptides to cancer cell membranes. Following membrane binding, DAA peptides kill cells by causing irreparable membrane damage and cell lysis whereas indirect-acting anticancer peptides enter the cytoplasm and cause cell death via the induction of apoptosis [4].

The unique mechanism of action of DAA peptides has several advantages over conventional chemotherapy. Unlike most chemotherapeutic agents, DAA peptides are broad-spectrum anticancer molecules that kill slow-growing and multidrug-resistant cancer cells [5,6,7]. In addition, DAA peptides synergize with chemotherapeutic drugs in vitro and in vivo, are well tolerated in vivo (at low to moderate concentrations) and attack primary tumors and metastases in tumor-bearing mice [5,7,8,9,10,11]. Importantly, certain DAA peptides also trigger antitumor immune responses that protect against tumor re-challenge, can be adoptively transferred to recipient mice, and contribute to the clearance of metastases [10,11]. Finally, because the anticancer activity of DAA peptides does not rely on unique receptors or specific signal transduction pathways [4], resistance to these peptides was previously thought unlikely to occur.

The purpose of this study was to use the pleurocidin-family DAA peptides NRC-03 and NRC-07 to generate and subsequently characterize DAA peptide-resistant cancer cells. Neither NRC-03 nor NRC-07 affected the viability of normal human fibroblasts and system administration of these DAA peptides did produce any adverse effects in treated mice [5]. We report that prolonged exposure of MDA-MB-231 breast cancer cells to increasing concentrations of two different DAA peptides, the pleurocidins NRC-03 and NRC-07, resulted in the generation of MDA-MB-231 variants that were refractory to the pleurocidins NRC-03 and NRC-07, as well as mastoparan. We show that peptide resistance correlated with decreased peptide binding to the cell membrane, and was associated with substantial alterations in cell morphology and cell membrane structure, suggesting that these DAA peptides share a common mechanism of membrane destruction, likely by interacting with similar, if not identical, cell-surface molecules. It is not completely clear what membrane molecules NRC-03 and NRC-07 target but it is likely to be molecules with negatively charged phospholipid head groups such as phosphatidylserine and phosphatidylglycerol [2,3,4]. DAA peptide-resistant MDA-MB-231 breast cancer cells maintained their susceptibility to chemotherapeutic drugs (tamoxifen, cisplatin, and paclitaxel), providing a strong rationale for combination therapy. Transcriptomic sequencing of parental and DAA peptide-resistant cells showed alterations in the expression of genes involved in angiogenesis, extracellular matrix (ECM) interactions, and antigen-processing and presentation. Importantly, we showed that NRC-03-and NRC-07-resistant MDA-MB-231 cells were unable to establish tumors in immune-deficient mice, suggesting that the changes required for decreased susceptibility to peptide-mediated cytotoxicity also significantly reduced tumorigenicity.

## 2. Materials and Methods

### 2.1. Cells and Cell Culture

MDA-MB-231 breast cancer cells were obtained from Dr. S. Drover (Memorial University of Newfoundland, NL, Canada). NRC-03-and NRC-07-resistant MDA-MB-231 cells were generated by continuous exposure of MDA-MB-231 cells to increasing concentrations of DAA peptides NRC-03 or NRC-07 for approximately one year. Peptide-resistant cells were capable of growing in the presence of 50 μM NRC-03 or NRC-07. However, treatment with DAA peptides NRC-03 or NRC-07 at greater than 50 μM resulted in excessive toxicity, indicating that only low-level resistance was generated. Parental MDA-MB-231 cells cultured for the same amount of time as resistant cells but in the absence of peptide were used as a control for all experiments. All cells were grown in Dulbecco’s Modified Eagle Medium (Sigma-Aldrich Canada, Oakville, ON, Canada) supplemented with 100 U/mL penicillin, 100 μg/mL streptomycin, 2 mM L-glutamine, 5 mM HEPES (pH 7.4), and 2.5% heat-inactivated fetal bovine serum (Invitrogen, Burlington, ON, Canada). Cells were seeded, in peptide-free medium, into tissue culture plates, and were cultured for 24 h to promote cell adhesion. Stock flasks were passaged as required to maintain optimal cell growth and were routinely confirmed to be free of mycoplasma contamination.

### 2.2. Reagents

Pleurocidin-family peptides NRC-03 (amino acid sequence: GRRKRKWLRRIGKGVKIIGGAALDHL-NH_2_) and NRC-07 (amino acid sequence: RWGKWFKKATHVGKHVGKAALTAYL-NH_2_) were synthesized by American Peptide Company (Sunnyvale, CA, USA) to > 95% purity by HPLC. Biotinylated NRC-03 and biotinylated NRC-07 (> 95% purity) were synthesized by Dalton Pharma Services (Toronto, ON, Canada), and were previously confirmed to be as cytotoxic as the non-biotinylated peptides^5^. Mastoparan (amino acid sequence: INLKALAALAKKIL-NH_2_) at > 95% purity was purchased from Peptide 2.0 Inc. (Chantilly, VA, USA). Sodium cacodylate, 3-(4,5-dimethylthiazol-2-yl)-2,5-diphenyltetrazolium bromide (MTT), cisplatin, docetaxel, and tamoxifen were from Sigma-Aldrich. Gluteraldehyde and osmium tetroxide were purchased from Electron Microscopy Sciences (EMS; Hatfield, PA, USA). Streptavidin-conjugated Texas Red fluorophore was purchased from Jackson Immunoresearch Laboratories (West Grove, PA, USA).

### 2.3. Animals

Highly immunodeficient adult (9-week-old) female NSG mice were bred and housed in the Modified Barrier Facility at the University of British Columbia (Vancouver, BC, Canada), and were maintained on a diet of sterilized rodent chow and water *ad libitum* Animal use was approved by the University of British Columbia Animal Care Committee and was in accordance with the Canadian Council of Animal Care guidelines.

### 2.4. MTT Assay

Breast cancer viability was determined using MTT assays [12], as previously described [5]. Percent cytotoxicity was calculated using the formula (1 − *E/C*) × 100), where *E* and *C* denote the absorbance of experimental and negative control samples, respectively.

### 2.5. Peptide Binding Assay

Peptide binding to parental, NRC-03-resistant, and NRC-07-resistant MDA-MB-231 breast cancer cells was assessed as previously described [5]. Slides were visualized using phase and UV microscopy, and fluorescence intensity was quantified using NIS-Elements software (Nikon Canada, Mississauga, ON, Canada).

### 2.6. Scanning Electron Microscopy

Parental, NRC-03-resistant, and NRC-07-resistant MDA-MB-231 breast cancer cells were seeded at 2 × 10^5^ cells/mL into 24-well flat-bottom tissue culture plates containing sterile coverslips and were cultured overnight to promote cell adhesion. The cells were fixed, dehydrated, dried to their critical point, mounted, and coated with gold as previously described [5]. The cells were viewed at the Institute for Research in Materials (Dalhousie University) on a Hitachi S4700 scanning electron microscope (Hitachi High Technologies, Rexdale, ON, Canada) at ×500, ×7000, and ×40,000.

### 2.7. RNA Sequencing Sample Preparation and Analysis

Parental, NRC-03-resistant, and NRC-07-resistant MDA-MB-231 cells were seeded into T25 tissue culture flasks and cultured until ~80% confluency of the monolayer was achieved. Cells were washed with phosphate-buffered saline (PBS) and then RNA was isolated using the Qiagen RNeasy Isolation kit (Qiagen, Valencia, CA, USA), according to manufacturer’s instructions. RNA concentration, integrity, and purity were assessed on the Agilent 2100 Bioanalyzer using the RNA Nano Kit (Agilent Technologies, Santa Clara, CA, USA). mRNA, which was purified from 1 mg of total RNA using poly-dT beads, was used for cDNA synthesis, followed by end repair, in which adaptors containing unique barcodes were added using 3′ end adenylation and ligation. Finally, DNA containing the adapter molecules was amplified by polymerase chain reaction and was then quantified. Cluster generation was carried out on a CBOT instrument followed by sequencing on a GAIIx instrument (Illumina, San Diego, CA, USA), which was performed as a single end run of 64 nucleotides. FASTQ files were demultiplexed using Illumina software (San Diego, CA, USA). TopHat2 [13] was used to align the reads to the Ensembl GRCh37.74 reference genome. SAMtools [14] was then used to sort and index the bam and sam files. Read count tables were generated using htseq-count (PMID: 25260700), and differential gene expression analysis was performed using edgeR [15]. Genes were deemed differentially expressed if they showed ≥ ± 1.5-fold change and had an adjusted *p*-value ≤ 0.05. Pathway over-representation analysis was performed using InnateDB (Vancouver, BC, Canada) [16], and protein-protein interaction network construction was performed using NetworkAnalyst (Vancouver, BC, Canada) [17], both of which were developed in the Hancock Laboratory. Note that in order to simplify networks, only genes with ≥ ± 2-fold change were used in network construction.

### 2.8. Breast Cancer Xenografts

A breast cancer xenograft model was used to test the ability of NRC-03-resistant and NRC-07-resistant cells to form tumors in mice. Briefly, groups of 5 NSG mice were injected with parental MDA-MB-231 cells, or with NRC-03-resistant or NRC-07-resistant MDA-MB-231 cells. Subcutaneous injection of tumor cells (5 × 10^6^ cells in 100 µL) was performed on the right hind flank in all mice. Mice were monitored every other day for tumor growth. Once tumors became palpable, caliper measurements were used to assess tumor volume over time. All mice were euthanized once the animals bearing parental tumors reached their humane endpoint (typically 30 days following cell injection). Tumors were excised, weighed, photographed, and fixed for histological examination.

### 2.9. Statistical Analysis

All data were analyzed by using the unpaired Student’s *t* test or one-way analysis of variance with the Bonferroni multiple comparison post-test, as appropriate.

## 3. Results

### 3.1. Continuous Exposure to Either NRC-03 or NRC-07 Results in Low-Level Resistance of Breast Cancer Cells to These Pleurocidins

**Figure 1:**
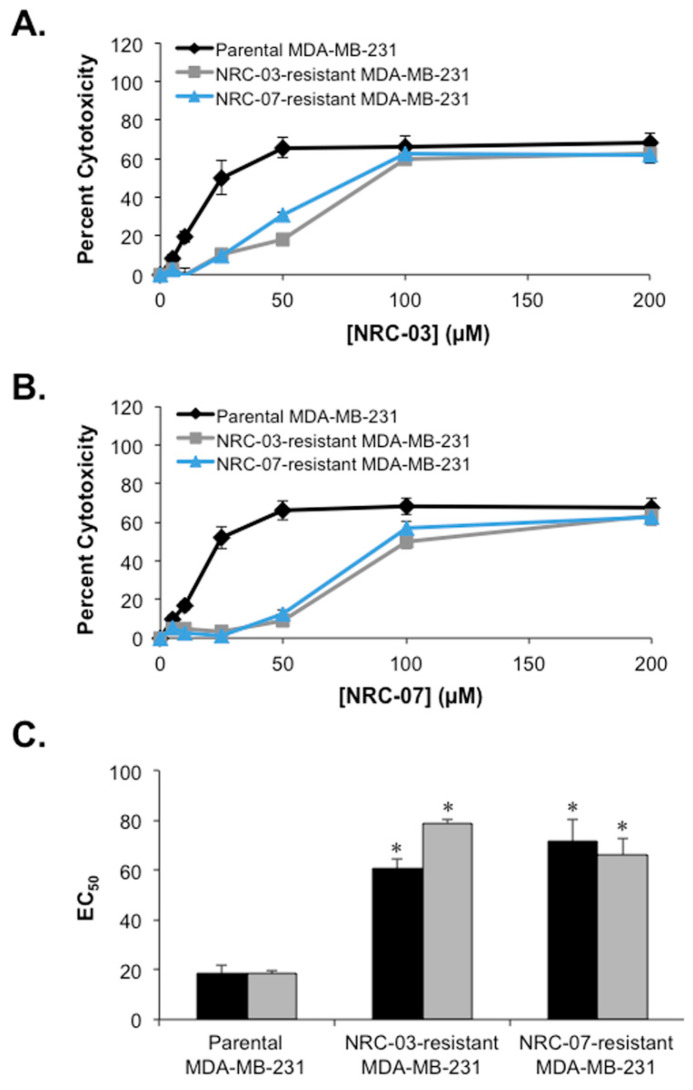
NRC-03-resistant and NRC-07-resistant breast cancer cells are refractory to both NRC-03 and NRC-07 in comparison to parental cells. Parental MDA-MB-231 cells, NRC-03-resistant MDA-MB-231 cells, and NRC-07-resistant MDA-MB-231 cells were cultured in the absence or presence of the indicated concentrations of (**A**) NRC-03 or (**B**) NRC-07 for 24 h. Cell viability was then determined by MTT assay. Data are significant (*p* < 0.0001) by ANOVA. (**C**) Cell viability measurements were used to calculate the EC_50_ of NRC-03 (black) and NRC-07 (grey). PBS was the vehicle for peptide. Data shown represent the mean of three independent experiments ± SEM and are statistically significant by the Bonferroni multiple comparisons test in comparison to parental MDA-MB-231 cells; * *p* < 0.01.

To generate NRC-03-resistant and NRC-07-resistant breast cancer cells, MDA-MB-231 cells were continuously cultured in the presence of increasing concentrations of the peptides NRC-03 or NRC-07. As a control, parental MDA-MB-231 cells were cultured, in parallel, in the absence of peptide. Cells were first exposed to 5 µM of each peptide. Peptide concentrations were not increased until the cells maintained their growth in the absence of cytotoxicity. After approximately one year of continuous exposure to NRC-03 or NRC-07, we obtained MDA-MB-231 cells that were able to grow in the presence of 50 µM peptide. Increasing the concentration of NRC-03 or NRC-07 beyond 50 µM resulted in excessive cell death. Dose-response experiments were performed to confirm resistance to NRC-03 and/or NRC-07. As shown in Figure 1, NRC-03-resistant and NRC-07-resistant cells were less susceptible to killing by both NRC-03 (Figure 1A) and NRC-07 (Figure 1B). The EC_50_ of NRC-03 for NRC-03-resistant and NRC-07-resistant cells increased by 3.3- and 3.8-fold, respectively (Figure 1C). Similarly, the EC_50_ of NRC-07 for NRC-03-resistant and NRC-07-resistant cells increased by 4.3- and 3.6-fold, respectively (Figure 1C). Cross-resistance to both NRC-03 and NRC-07 suggests that these DAA peptides share a common mechanism of action.

### 3.2. NRC-03-Resistant and NRC-07-Resistant Breast Cancer Cells Are Susceptible to Chemotherapeutic Drugs but Refractory to an Unrelated DAA Peptide

**Figure 2:**
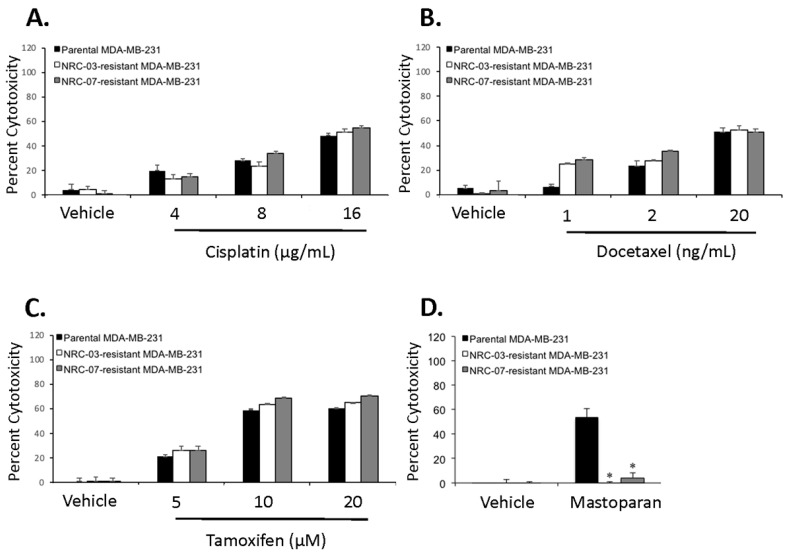
NRC-03-and NRC-07-resistant breast cancer cells are killed by cisplatin, docetaxel, and tamoxifen, but not mastoparan. Parental MDA-MB-231 cells, NRC-03-resistant MDA-MB-231 cells, and NRC-07-resistant MDA-MB-231 cells were cultured in the absence or presence of the indicated concentrations of (**A**) cisplatin, (**B**) docetaxel, (**C**) tamoxifen, or (**D**) mastoparan (25 μM). Cell viability was determined by MTT assay after 24 h (mastoparan) or 72 h (cisplatin, docetaxel, and tamoxifen). PBS was the vehicle for cisplatin and mastoparan, and dimethyl sulfoxide (<0.01%) was the vehicle for docetaxel and tamoxifen. Data shown represent the mean of technical replicates ± standard deviation and are representative of 2–3 independent experiments. Statistical significance, in comparison to parental MDA-MB-231 cells, was determined by the Bonferroni multiple comparisons test (* *p* < 0.0005).

We next tested whether resistance to the DAA peptides NRC-03 and NRC-07 also conferred resistance to chemotherapeutic drugs. As shown in Figure 2, we found that NRC-03-resistant and NRC-07-resistant MDA-MB-231 cells were as susceptible as parental cells to killing by cisplatin (Figure 2A), docetaxel (Figure 2B), and tamoxifen (Figure 2C). The calculated EC_50_ for cisplatin was 16.2 for parental cells, 16.0 for NRC-03-resistant cells, and 13.9 for NRC-07-resistant cells. The calculated EC_50_ for docetaxel was 19.1 for parental cells, 17.9 for NRC-03-resistant cells, and 18.6 for NRC-07-resistant cells. The calculated EC_50_ for tamoxifen was 13.6 for parental cells, 12.5 for NRC-03-resistant cells, and 11.4 for NRC-07-resistant cells. We also determined whether NRC-03-resistant and NRC-07-resistant cells were susceptible to killing by mastoparan, an unrelated 14-residue DAA peptide isolated from wasp venom [18]. Figure 2D shows that both NRC-03-resistant and NRC-07-resistant cells were refractory to the cytolytic activity of mastoparan, suggesting that pleurocidins and mastoparan share a common mechanism of action.

### 3.3. Breast Cancer Cell Resistance to NRC-03 and NRC-07 Is Associated With Reduced Peptide Binding and Altered Cell Morphology

**Figure 3:**
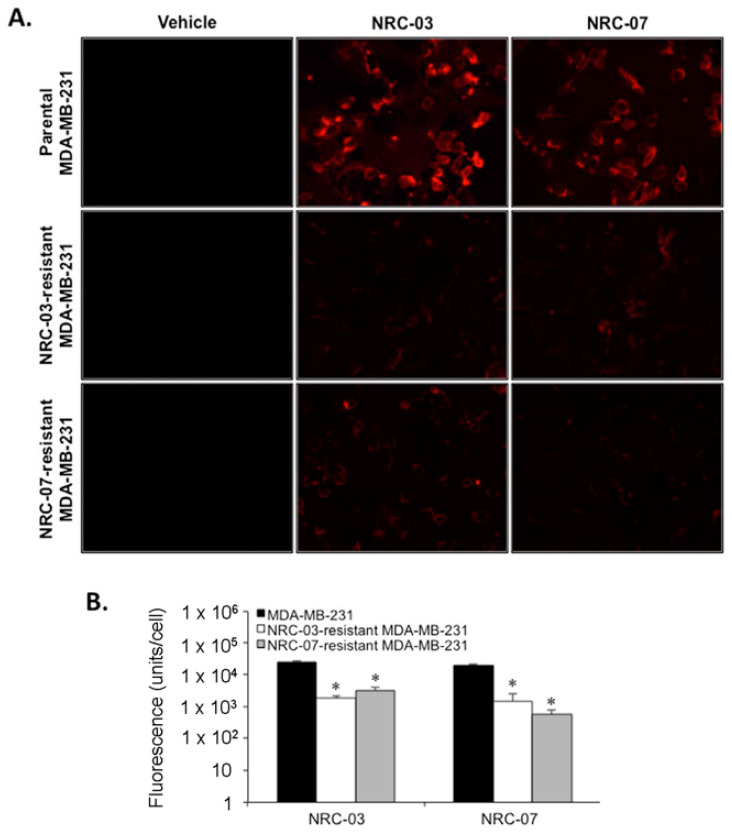
NRC-03 and NRC-07 bind poorly to NRC-03-resistant and NRC-07-resistant breast cancer cells. (**A**) Parental MDA-MB-231 cells, NRC-03-resistant MDA-MB-231 cells, and NRC-07-resistant MDA-MB-231 cells were cultured in the absence or presence of 50 µM biotinylated NRC-03 or biotinylated NRC-07 for 10 min, stained with Texas Red-streptavidin, and visualized by fluorescence microscopy at 20×. (**B**) Peptide binding was quantified using NIS-Elements. The vehicle for peptides was PBS. Data shown represent the mean of three independent experiments ± SEM. Statistical significance was determined by the Bonferroni multiple comparisons test in comparison to parental MDA-MB-231 cells; * *p* < 0.001.

**Figure 4:**
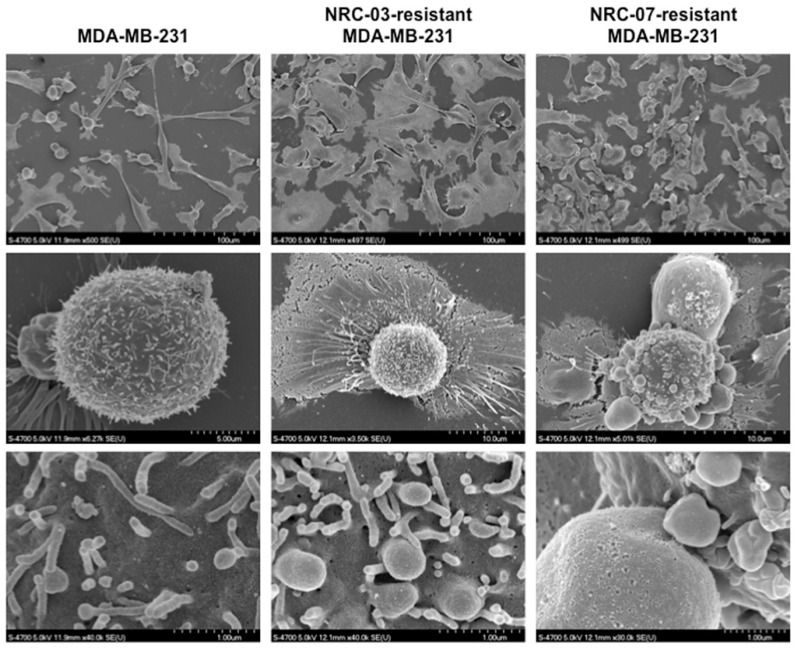
NRC-03-resistant and NRC-07-resistant breast cancer cells are visually distinct from parental cells. Parental MDA-MB-231 cells, NRC-03-resistant MDA-MB-231 cells, and NRC-07-resistant MDA-MB-231 cells were grown overnight on circular coverslips. Cellular ultrastructure was visualized by scanning electron microscopy. Data shown are from a representative experiment (n = 2).

Since cationic DAA peptides preferentially bind to cancer cells on the basis of electrostatic interactions, owing to the fact that cancer cell membranes carry a net negative charge due to increased levels of several different anionic surface molecules [2,3,4], we anticipated that NRC-03-resistant and NRC-07-resistant MDA-MB-231 cells would bind less NRC-03 and NRC-07. As expected, binding of fluorophore-labeled NRC-03 and NRC-07 to peptide-resistant cells was significantly reduced (Figure 3). Binding of NRC-03 to NRC-03-resistant and NRC-07-resistant cells was decreased by 14-fold and 8-fold, respectively. NRC-07 binding to NRC-03-resistant and NRC-07-resistant cells was reduced by 14-fold and 35-fold, respectively. Scanning electron microscopy showed that the cell membrane of NRC-03-resistant and NRC-07-resistant cells was intact (Figure 4); however, resistance to NRC-03 and NRC-07 was associated with marked alterations in cell morphology. NRC-03-resistant cells were more cuboidal in shape and were not as spindled in appearance as parental cells. Moreover, the few cells with rounded cell bodies (e.g., middle panel) showed abundant membrane projections in comparison to parental cells. High magnification showed membrane blebbing that was not observed in parental cells. In contrast, NRC-07-resistant cells maintained their spindled morphology; however, these cells showed extensive membrane blebbing that could be observed at low magnification (left panels; 500×), intermediate magnification (middle panels; 35,000–63,000×), and high magnification (right panels; 300,000–400,000×). Membrane blebbing in NRC-03-resistant and NRC-07-resistant cells was not a consequence of apoptosis since all cells were > 95% viable, as assessed by MTT and trypan blue dye exclusion viability assays. 

### 3.4. NRC-03-Resistant and NRC-07-Resistant Breast Cancer Cell-Derived Xenografts Exhibit Impaired Growth and Angiogenesis in Immune-Deficient Mice

**Figure 5:**
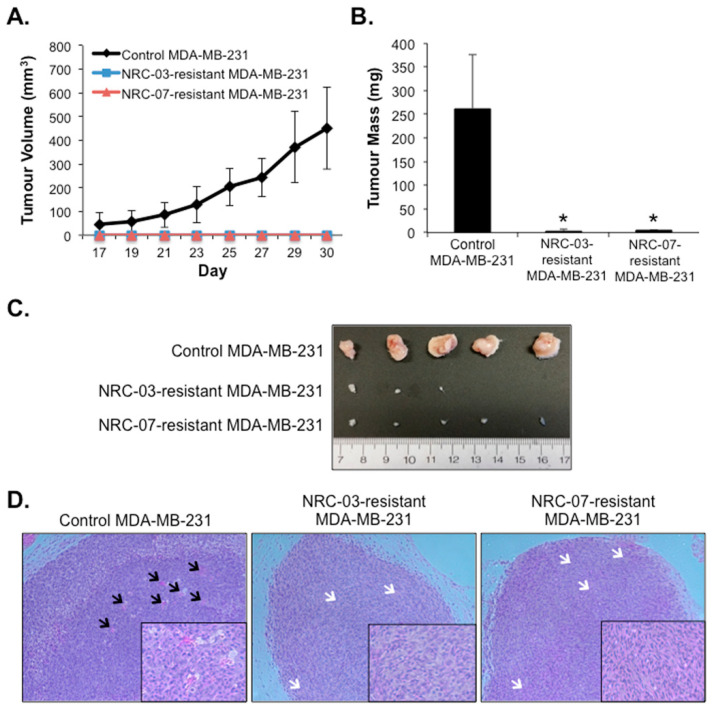
NRC-03-resistant and NRC-07-resistant breast cancer cells exhibit impaired tumorigenicity, tumor growth and tumor-associated angiogenesis. Parental MDA-MB-231 cells, NRC-03-resistant MDA-MB-231 cells, or NRC-07-resistant MDA-MB-231 cells were implanted by subcutaneous injection of 5 × 10^6^ cells into the hind flank of NSG mice. (**A**) Tumor volume was recorded by caliper measurements every other day until the first mouse reached its humane endpoint, at which time the mice were euthanized, and excised tumors were (**B**) weighed, and (**C**) photographed. (**D**) Tumors were fixed, sectioned and stained with hematoxylin and eosin. Images shown are from representative mice and were captured at 10× and 20× (inset) magnification. Black and white arrows indicate blood vessels and striated tumor tissue, respectively. Data shown in panel A and panel B represent the average of 5 mice ± SD and are significant (* *p* < 0.0001) by the Bonferroni multiple comparisons test. Note that NRC-03-resistant and NRC-07-resistant tumors were palpable on day 19, but could not be accurately measured; hence, statistical analyses were not performed on data depicted in panel A.

To further assess the therapeutic implications of cancer cell resistance to DAA peptides, we compared the tumorigenicity of NRC-03-resistant and NRC-07-resistant MDA-MB-231 cells to that of parental cells. Despite their advanced passage number, tumors arising from parental MDA-MB-231 breast cancer cells became palpable 17–19 days following implantation (100% tumor take) and grew at the same rate as previously reported [5]. Tumors arising from NRC-03-resistant and NRC-07-resistant cells also became palpable 17–21 days following implantation (67% and 100% tumor take, respectively). However, none of the tumors derived from peptide-resistant cells grew large enough that tumor volume could be reliably determined by caliper measurements (Figure 5A). Excised tumors derived from NRC-03-resistant and NRC-07-resistant cells weighed significantly less and were substantially smaller than parental tumors (Figure 5B,C, respectively). In contrast to parental tumors, hematoxylin and eosin staining showed that blood vessels (indicated by black arrows) were absent in tumors arising from NRC-03-resistant and NRC-07-resistant cells. Interestingly, while the cells of tumors arising from parental MDA-MB-231 cells exhibited round nuclei, normal cell shape, and typical cellular organization, cells of tumors arising from the NRC-03-resistant and NRC-07-resistant MDA-MB-231 cells were spindled, had elongated nuclei, and were aligned in random smeared striations (indicated by white arrows, Figure 5D). Collectively, these data suggest that the acquisition of resistance to these DAA peptides negatively impacts cell characteristics that are required for MDA-MB-231 tumorigenicity and tumor growth.

### 3.5. Resistance to NRC-03 and NRC-07 Is Associated With Altered Expression of Genes Involved in ECM Organization and Angiogenesis

**Figure 6:**
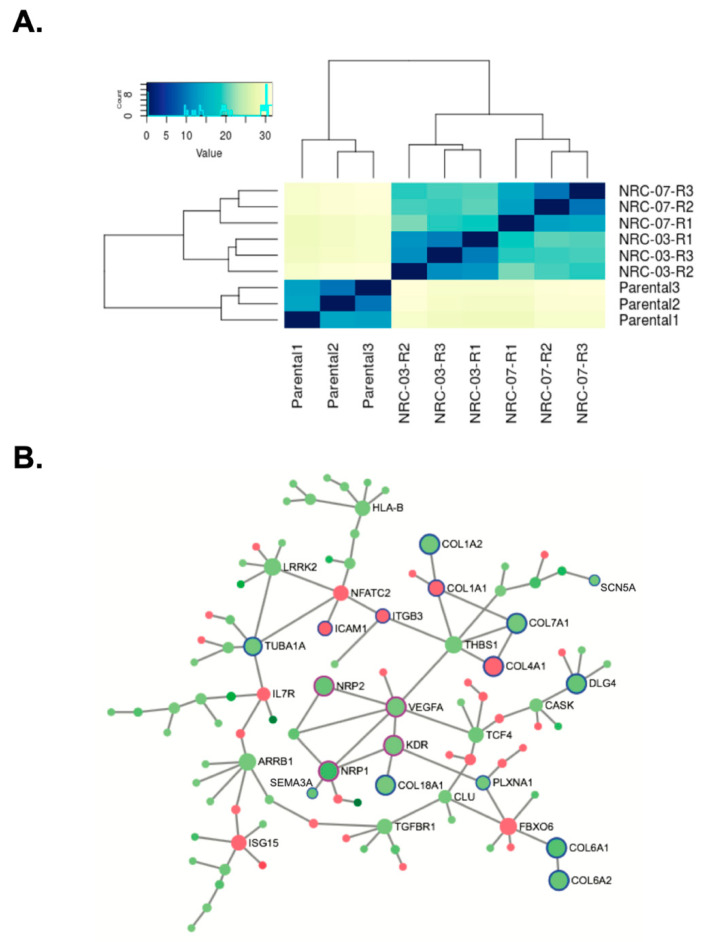
Breast cancer cell resistance to NRC-03 and NRC-07 is associated with the differential expression of genes involved in angiogenesis and ECM interaction-related pathways. (**A**) RNA sequencing analysis was performed on parental MDA-MB-231 cells, NRC-03-resistant MDA-MB-231 cells, and NRC-07-resistant MDA-MB-231 cells. The heat map compares the three cell lines. The color key and histogram are in the upper left quadrant. R1, R2 and R3 refer to distinct biological replicates. (**B**) A zero-order interaction network of differentially expressed genes between parental cells and peptide-resistant cells was prepared using NetworkAnalyst [17]. Up-regulated and down-regulated genes are shown as red and green filled circles, respectively. Up-regulated and down-regulated genes involved in angiogenesis, antigen processing and presenting, and ECM interactions are outlined in purple and blue, respectively.

RNA-Seq analysis was used to gain insights into the possible reason(s) for the impaired growth of tumors formed by NRC-03-resistant and NRC-07-resistant MDA-MB-231 cells. A heat map and hierarchical clustering of samples showed that NRC-03-resistant and NRC-07-resistant cells were more similar to each other than they were to parental cells (Figure 6A). Since resistance to one DAA peptide conferred resistance to both a related (pleurocidin-family) and an unrelated (mastoparan) DAA peptide, we were interested in common differences in gene expression between the peptide-resistant and parental cells. There were 973 genes (greater than or equal to 1.5 absolute fold-change, adjusted *p*-value less than or equal to 0.05) that were differentially expressed by parental and peptide-resistant cells; 263 and 710 genes that were up-regulated and down-regulated, respectively. Signature over-representation analysis was used with the Reactome gene annotation system to identify pathways that were enriched among up-regulated or down-regulated genes in peptide-resistant cells. This analysis technique identified 18 up-regulated pathways (adjusted *p*-value less than or equal to 0.05) in peptide-resistant cells (Table 1). Of particular interest were those genes involved in extracellular matrix (ECM) interactions. Other differentially expressed genes encode proteins involved in the suppression of angiogenesis, induction of tumor cell apoptosis, and decreased tumorigenicity, including *COL4A1* (arresten), *COL4A2* (canstatin), and *COL4A3* (tumstatin). *LOX* and *LOXL3,* which encode oxidases that cause post-translational oxidative deamination of lysine residues in collagen and elastin, were also up-regulated in these pathways. Signature over-representation analysis identified 29 down-regulated pathways in peptide-resistant cells (Table 2). Biologically relevant down-regulated genes included those involved in ECM-related pathways, vascular endothelial growth factor signaling, O-linked glycosylation, glycosaminoglycan metabolism, chondroitin sulfate/dermatan sulfate metabolism, and heparan sulfate/heparin metabolism. Many of these differentially expressed genes also fit into a zero-order interaction network (Figure 6B), suggesting the dysregulation of dozens of directly interacting proteins. This network contained significantly large numbers of proteins that participate in angiogenesis and ECM organization.

## 4. Discussion

This study shows for the first time that prolonged and continuous exposure of MDA-MB-231 human breast cancer cells to the pleurocidin-family DAA peptides NRC-03 and NRC-07 resulted in the generation of variants with low-level resistance to both of these DAA peptides, as well as the unrelated 14-residue wasp-derived DAA peptide mastoparan. Cross-resistance to different DAA peptides suggests that these DAA peptides target and damage cancer cells by a similar mechanism. Importantly, DAA-resistant cancer cells retained their susceptibility to the cytotoxic chemotherapy drugs cisplatin (alkylating agent) and docetaxel (antimitotic), as well as the estrogen receptor antagonist tamoxifen that also induces apoptosis in estrogen receptor-negative breast cancer cells via inhibition of cancerous inhibitor of protein phosphatase 2A [19]. Cancer cell resistance to NRC-03 and NRC-07 was associated with reduced binding of peptide to the cell membrane and a distinctly different appearance in comparison to the parental cells; notably, with more membrane blebbing and visible pore formation for the NRC-03-and NRC-07-resistant cells compared to the parental cells. This suggests that resistance was mediated by altered membrane composition that did not compromise cellular susceptibility to chemotherapeutic compounds that do not directly disrupt the cell membrane.

Cationic DAA peptides such as NRC-03, NRC-07 and mastoparan interact with anionic structures such as phosphatidylserine, heparan sulfate proteoglycans, chondroitin sulfate proteoglycans and sialylated glycoproteins [5,20,21,22,23]. However, abolishing peptide binding to any one class of these molecules does not prevent peptide-mediated cell death, suggesting that cationic DAA peptides lack a unique receptor, and are rather attracted to several different classes of anionic cell surface molecules. Further to this, cross-resistance to different DAA peptides suggests that the interaction between these DAA peptides and cancer cell membranes is mediated by a common collection of negatively charged cell-surface molecules. Therefore, by comparing parental and DAA peptide-resistant breast cancer cells, we were uniquely poised to further investigate the mechanism of DAA attraction to cancer cell membranes. To this end, RNA-Seq analysis of peptide-resistant cells showed down-regulation of genes involved in O-linked glycosylation, as well as chondroitin and heparan sulfate proteoglycan metabolism. All of these genes are associated with the expression of negatively charged structures on the cell surface that are known to be involved in DAA peptide-mediated cytotoxicity [5,20,21,22,23].

Signature over-representation analysis also revealed the down-regulation of genes involved in several ECM-related pathways, including genes that encode collagens, glycoproteins involved in cell–cell and/or cell–ECM interactions, several different matrix metalloproteases, proteins involved in vascular development, ECM degradation, growth and migration, proteins that control cell shape, size, and mobility, components of the basement membrane, regulators of cellular differentiation and cell death, and proteins involved in blood vessel development. Collectively, these alterations are likely to completely restructure the cellular membrane and, consequently, the cell–cell and cell–ECM interactions that are required for tumor growth and survival. As a consequence of these alterations, it is not surprising that DAA peptide-resistant tumors exhibited marked differences in cell shape and organization.

The tumorigenicity of NRC-03- and NRC-07-resistant MDA-MB-231 cells was assessed to further address the therapeutic implications of cancer cell resistance to cytolytic peptides. While all established tumors became palpable at the same time, none of the tumors derived from DAA peptide-resistant cells underwent appreciable growth. Histologic examination of tumor sections revealed the complete absence of vasculature in tumors derived from NRC-03-and NRC-07-resistant MDA-MB-231 cells. In addition to the dysregulation of genes involved in ECM organization, RNA-Seq analysis showed down-regulation of the vascular endothelial growth factor receptor pathway in DAA peptide-resistant cells. Furthermore, the angiogenesis inhibitors *COL4A1* (arresten), *COL4A2* (canstatin), and *COL4A3* (tumstatin) were up-regulated. Collectively, these findings suggest a tumor environment wherein the ECM is altered, angiogenesis is inhibited, O-linked oligosaccharide biosynthesis is impaired, and chondroitin and heparan sulfate metabolism is inhibited. While certain cationic anticancer peptides such as lactoferricin inhibit angiogenesis by competing for heparin-like binding sites on endothelial cells [24], we still do not understand the relationship between NRC-03-and NRC-07-mediated cytotoxicity and angiogenesis.

## 5. Conclusions

Our findings, in combination with our previous reports [5,8,22], suggest that DAA peptides such as NRC-03 and NRC-07 are excellent candidates as adjunctive therapies for the treatment of cancer since these cytolytic peptides act as chemosensitizing agents, kill slow-growing and multidrug-resistant cancer cells, are exceedingly difficult to develop resistance to, and, when resistance does occur, tumorigenicity is severely impaired and the DAA peptide-resistant cancer cells remain susceptible to chemotherapy. In the future, it will be important to determine whether palpable tumors, derived from DAA peptide-resistant MDA-MB-231, and other breast cancer cell lines can be completely eradicated by treatment with chemotherapeutic compounds, as well as the role that the ECM plays in DAA peptide-mediated cytotoxicity.

## Figures and Tables

**Table 1:** Up-regulated pathways and genes in peptide-resistant MDA-MB-231 breast cancer cells.

Pathway	Adjusted *p*-Value	DE Genes in Pathway (Weighted)	Pathway Size	Genes
Molecules associated with elastic fibres	*p* < 0.001	23	728	ITGB3;FN1;LTBP3;EFEMP2;FBN1;SERPINH1;PLOD2;MATN3;ADAM12;IL18;OLR1;MMP19;MMP15;COL12A; ICAM1;CDC14B;CXADR;IL7R;SDC4;LUM;COL5A2;COL4A4;COL4A2;COL4A3
Cell-ECM interactions	*p* < 0.001	10	72	LIMS2;FERMT2;FNLC;FBLIM1;LIMS1
VEGF receptor 2-mediated cell proliferation	*p* < 0.001	15	365	PRKZ2;SPHK1;DUSP1;SPTB;RASGRP3;RASA4;FN1;KBTBD7;ITPR2;STPBN2;IL6R
Gap junction trafficking and regulation	*p* < 0.001	10	211	TUBA4A;TUBB6;SRF;TTC21B;DIAPH3;FMNL2;RHOD;DAAM1;TTBK2;BORA;IQCB1;PROS1;RHOB;RAB3IP; FN1;ITGB3
Downstream signaling of activated FGF receptor 1	*p* < 0.001	14	570	PEA15;CNKSR1;CSF2RA;TNFC6C;RASA4;FN1;ITPR2;FOXO1;DUSP8;ITGB3;PRKCZ;SPHK1;IL6R
Signalling to RAS	*p* < 0.001	13	597	FN1;ITGB3;DUSP8;RASA4;KBTBD7;PRKCZ;SPHK1;DUSP16;PROS1;COL5A2
Signaling by Interleukins	*p* < 0.001	14	620	IL18;IL7R;IL1A;IL6R;RAPGEF1;GAB2;SPTB;RASA4;DUSP16;DUSP1
SHC1 events in EGF receptor signaling	*p* < 0.001	8	222	CSF2RA;CNKSR1;DUSP8;DUSP16;SPTBN2;RAPGEF1;RIT1
Signalling to p38 via RIT and RIN	*p* < 0.001	6	86	RIT1;SPTBN2;DUSP16;RAPGEF1;RASGRP3;PEA15;DUSP1;KBTBD7
Assembly of collagen fibrils and other multimeric structures	*p* < 0.001	8	165	LOX;LOXL3;COL5A2;COL4A4;COL4A3;COL1A1;COL4A2;COL4A1
RHO GTPases activate formins	*p* < 0.001	12	683	SRF;DIAPH3;FMNL2;RHOD;DAAM1RHOB
Collagen degradation	*p* = 0.002	15	1545	COL12A1;MMP19;MMP15;COL5A2;COL4A4;COL4A3;COL4A2;COL4A1;COL1A1
ARMS-mediated activation	*p* = 0.003	8	398	SPTB;RASA4;FN1;ITGB3RAPGEF1;RIT1
RHO GTPase effectors	*p* = 0.003	21	3014	RHPN2;CTTN;PKN3;AB12;BAIAP2;SRF;DIAPH3;FMNL2RHOD;MYL9;MYL12B;TUBB6;RHOB
NOD1/2 signaling pathway	*p* = 0.006	4	17	CASP4;CASP2;CYLD;TNFAIP3;BIRC3
Signaling by FGFR1	*p* = 0.008	17	2231	ITGB3;FOXO1;CSF2RA;SPTB;RASA4;KBTBD7;SPTBN2;DUSP16;DUSP1;DNAL4;ABI2;BAIAP2;IL6R;STAT1; CDC14B;MATN3;ICAM1;FBNM1;NCOA3
Downstream signaling of activated FGF receptor 4	*p* = 0.026	13	1508	FN1;CNKSR1;PEA15;NR4A1;TNRC6C;RASA4;DUSP!;ITPR2;FOXO1;DNAL4;MATN3;ABI2;BAIAP2;IL6R;STAT1; ICAM1;MMP19;RIT1;MMP15
Effects of PIP2 hydrolysis	*p* = 0.036	11	1129	DGKA;DGKH;MGLL;ITPR2;PRKCz;ADAM12;PROS1;RHOB;GAB2;PLCG2;TUBA4A;SPTB;RASA4;DUSP16; DUSP1;KBTBD7;RASGRP3;SPTBN2;DUSP8

**Table 2:** Down-regulated pathways and genes in peptide-resistant MDA-MB 231 breast cancer cells.

Pathway	Adjusted *p*-Value	DE Genes in Pathway (Weighted)	Pathway Size	Genes
ECM organization	*p* < 0.001	78	3507	MUSK;PTPRS;LRP4;NTN4;LTBP1;LTBP4;LTBP2;BMP4;GDF5;ADAM15;ADAM8;COL27A1;MMP16;CASK; ITGA3;MMP14;KDR;ITGA7;ADAMTS9;CTSD;ITGB8;TIMP1;TNC;THBS1;COL8A1;COL7A1;COL18A1;HSPG2; COL6A1;COL6A2;COL5A1;COL4A5;COL1A2
L1CAM interactions	*p* < 0.001	39	991	CNTNAP1;NRP2;MSN;ANK1;NRCAM;ANK2;SCN9A;SCN5A;EPHB2;DPYSL2;KIF4A;DLG3;DLG1;NRP1; RPS6KA5;TUBA1A;DLG4;KCNQ3
Asymmetric localization of PCP proteins	*p* < 0.001	15	93	SMURF2;PRICKLE1;FZD7;FZD1;FZD8;FZD2
Signaling by VEGF	*p* < 0.001	25	406	PGF;NRP2;VEGFC;VEGFA;NRP1;AXL;CYFIP2;KDR;WASF3;CYBA;RASGRF1;DUSP4;EREG;DUSP6;DUSP10; IL17RD;RASA1;CNKSR2;ANGPT1;NRG1;IRS1;ARRB1;EGF;DLG4
Rho GTPase cycle	*p* < 0.001	82	4631	OPHN1;ARHGAP5;CHN2;RHOU;RHOJ;ARHGAP22;STARD8;ARHGAP29;ARHGAP24;FAM13A;ARHGAP26; FGD1;ARHGEF4;NET1;ARHGEF6
Downregulation of SMAD2/3:SMAD4 transcriptional activity	*p* < 0.001	24	425	NEDD4L;SMURF2;HDAC1;SMAD3;TGIF1;UBE2A;ASB9;DET1;SPSB1;RNF182;PJA1;ASB13;RNF43;ANO2; ANO8;NALCN;CLCN3;SCNN1A;CLCN2;SCNN1D;ANO5;TPCN1;DZIP1;WWTR1;RCHY1;SOX4;SOX9;GPR161; CTNNBIP1;PRKG1;UBE2L6;NGFRAP1;DNER;CDON;CAV1;SLC25A5;GNAO1;FGD1;ARHGEF4;TLE3;NET1; XIAP;ARHGEF6;FZD1;FZD8;FZD2;NGFR;AMER1;CDC20;H2AFZ;ARRB1
O-glycosylation of TSR domain-containing proteins	*p* < 0.001	26	618	SEMA5A;ADAMTS12;SPON2;ADAMTS9;ADAMTS15;ADAMTSL1;THBS2;THBS1
O-linked glycosylation	*p* < 0.001	31	909	GALNT18;GALNT12;GALNT5;GALNT14;C1GALT1C1;B3GNT5;ADAMTS12;SPON2;ADAMTS15;ADAMTSL1; THBS2;SEMA5A;ADAMTS9;B3GNT7;CFP;THBS1;ST6GAL1;ST3GAL4
Signaling by platelet derived growth factor	*p* < 0.001	22	499	PDGFD;PLAT;THBS2;THBS1;PDE1C;CAMK4;COL6A1;COL6A2;COL5A1;COL4A5;RASGRF1;DUSP4;EREG; DLG4;DUSP6;DUSP10;IL17RD;RASA1;CNKSR2;ANGPT1;NRG1;IRS1;ARRB1;EGF
Collagen degradation	*p* < 0.001	37	1545	CTSD;COL6A1;COL8A1;COL13A1;COL6A2;COL5A1;MMP14;COL18A1;COL7A1;COL4A5;COL1A2
Degradation of the extracellular matrix	*p* < 0.001	32	1242	ADAM15;ADAM8;MMP16;ADAMTS9;TIMP1;CTSS;CTSD;COL13A1;HSPG2;MMP14;COL8A1;COL7A1; COL6A1;COL6A2;COL5A1;COL4A5;COL1A2;COL18A1
Downstream signaling of activated FGF receptor 1	*p* < 0.001	21	570	PRKAR2B;PIK3R3;CAMK4;DUSP6;IRS1;DUSP4;EREG;DLG4;DUSP10;IL17RD;RASA1;CNKSR2;PDE1C;NRG1; NRG2;GNAO1;KDR;SYNJ2;PIK3CG;AJUBA;NPHP4;SPRY2;PI4K2B;DLG3;GRIK4;GRIK2;DLG1;GNB4
Interaction between L1 and ankyrins	*p* < 0.001	13	219	ANK1;NRCAM;ANK2;SCN9A;SCN5A;KCNQ3
Glycosaminoglycan metabolism	*p* < 0.001	29	1239	HAS2;PAPSS1;HS3ST1;HS3ST3B1;NDST3;B3GNT7;NAGLU;UST;CHST11;GXYLT2;CHST15;EXT1;ST3GAL4; IDS;IDUA;CSPG4;HSPG2
Chondroitin sulfate/dermatan sulfate metabolism	*p* < 0.001	17	450	HSPG2;UST;GXYLT2;CHST11;CHST15;IDS;IDUA;CSPG4;HS3ST1;HS3ST3B1;NDST3;NAGLU;EXT1
Hexose transport	*p* < 0.001	7	62	SLC2A1;PGLS;SORD;SLC4A7;SLC29A1;SLC9A5;SLC29A3;SLCO4A1;GALK1;SLC16A10;PFKFB4;KHK;PFKL; G6PD;HAS2;HS3ST1;ENO1;UST;CHST11;CHST15;SLC7A5;PAPSS1;SLC1A1;NAGLU;B3GNT7;IDS;GYG2; SLC2A1;PGLS;SORD;SLC4A7;SLC29A1;SLC9A5;SLC29A3;SLCO4A1;GALK1;SLC16A10;PFKFB4;KHK;PFKL;G6PD; HAS2;HS3ST1;ENO1;UST;CHST11;CHST15;SLC7A5;PAPSS1;SLC1A1;NAGLU;B3GNT7;IDS;GYG2;ST3GAL4; HSPG2;PRKAA2ST3GAL4;HSPG2;PRKAA2
Signaling by EGF receptor	*p* < 0.001	16	428	LRIG1;SPRY1;SH3KBP1;PDE1C;CAMK4;RASGRF1;DUSP4;DLG4;ARRB1;DUSP6;DUSP10;IL17RD;RASA1; CNKSR2;ANGPT1;EREG;NRG1;IRS1;SPRY2;EGF
O-linked glycosylation of mucins	*p* < 0.001	26	1231	GALNT18;GALNT12;GALNT5;GALNT14;C1GALT1C1;B3GNT5;B3GNT7;ST6GAL1;ST3GAL4
Collagen formation	*p* = 0.001	28	1336	CTSS;COLGALT2;LOXL4;COL13A1;MUSK;PTPRS;LRP4;NTN4;LTBP1;LTBP4;LTBP2;BMP4;GDF5;ADAM15; ADAM8;ITGA7;CASK;ITGA3;TNC;MMP16;CD74;CTSH;CTSC;CTSF;KDR;MMP14;ADAMTS9;ITGB8;THBS1; TIMP1;CTSD;HSPG2;UBE2A;ASB9;DET1;ERAP1;SPSB1;RNF182;PJA1;ASB13;KIF4A;KIF3C;RCHY1;DYNC2H1; SIGIRR;IRAK3;NEDD4L;CENPE;SMURF2;PELI2;MEF2C;RPS6KA5;CDC20;ELK1;TAB3;DUSP4;DUSP6;TUBA1A
VEGF receptor 2-mediated cell proliferation	*p* = 0.001	14	365	ITPR1;KDR;IL17RD;ANGPT1;DLG4;NRG2;RASGRF1;DUSP4;ARRB1;EGF;GNAO1;PPP3CA;SPTBN5;SPRY2; PDE3B;PLA2G4A
The activation of arylsulfatases	*p* = 0.006	6	66	ARSD;ARSE;ARSJ;ARSI
HS-GAG biosynthesis	*p* = 0.006	10	236	HS3ST1;HS3ST3B1;NDST3;EXT1;HSPG2
MyD88:Mal cascade initiated on plasma membrane	*p* = 0.006	8	101	IRAK3;SIGIRR;SAA1;MEF2C;RPS6KA5;ELK1;TAB3;DUSP4
Post-translational protein modification	*p* = 0.012	106	9940	PIGA;MAN1A1;ARSD;ARSE;ARSJ;ARSI;PHC1;PHC2;FUT8;GAS6;ALG6;ALG13;ST3GAL5;ST6GALNAC5;MGAT4A; GALNT18;GALNT12;GALNT5;ADAMTS12;GALNT14;SPON2;ADAMTS15;C1GALT1C1;B3GNT5;ADAMTSL1;MPI; STAG2;ST8SIA4;THBS2;SRD5A3;AAAS;SEC31A;SEMA5A;ADAMTS9;CALR;B3GNT7;CFP;THBS1;ST6GAL1;ST3GAL4
Heparan sulfate/heparin (HS-GAG) metabolism	*p* = 0.013	26	1440	HS3ST3B1;NDST3;EXT1;GXYLT2;IDUA;CSPG4;PGLS;SORD;PFKFB4;PFKL;G6PD;KHK;ENO1;GALK1;SLC25A10; SLC2A1;GYG2;AAAS;ALG6;ALG13;MPI;SRD5A3;HLCS;CYP2U1;FDXR;TBXAS1;ACACB
Ethanol oxidation	*p* = 0.024	8	169	ALDH2;ACSS1;CYP2U1;PTGS1;GLUL;ALDH5A1;CYP2J2;FDXR;TBXAS1;BCHE;CYP39A1;DLG3;GRIK4;GRIK2; CACNB3;PPFIA4;CASK;DLG1;RIMS1;GNB4;CAMK4;DLG4
Constitutive Signaling by EGF receptor vIII	*p* = 0.031	4	27	EGF;LAMP2;CLU;KDR;VEGFC;TIMP1;GAS6;VEGFA;THBS1;SPRY2
Glycerophospholipid biosynthesis	*p* = 0.042	29	1731	PNPLA3;CDS1;PEMT;DGAT2;LPCAT2;MBOAT1;SLC44A5;PLB1;BCHE;GPAT3;GPD1L;PLA2G12A;PLA2G4A
Integrin cell surface interactions	*p* = 0.046	16	712	ITGB8;KDR;ITGA3;THBS1;TNC;COL13A1;ITGA7;COL8A1;COL6A1;COL6A2;COL7A1;COL18A1;COL5A1; COL1A2;COL4A5;HSPG2
